# Plasmonic Superstructure Arrays Fabricated by Laser Near-Field Reduction for Wide-Range SERS Analysis of Fluorescent Materials

**DOI:** 10.3390/nano12060970

**Published:** 2022-03-15

**Authors:** Shi Bai, Anming Hu, Youjin Hu, Ying Ma, Kotaro Obata, Koji Sugioka

**Affiliations:** 1 Advanced Laser Processing Research Team, RIKEN Center for Advanced Photonics, 2-1 Hirosawa, Wako, Saitama 351-0198, Japan; shi.bai@riken.jp (S.B.); kobata@riken.jp (K.O.); 2 Department of Mechanical, Aerospace and Biomedical Engineering, University of Tennessee Knoxville, 1512 Middle Drive, Knoxville, TN 37996, USA; ahu3@utk.edu; 3 Institute of Laser Engineering, Faculty of Materials and Manufacturing, Beijing University of Technology, 100 Pingle Yuan, Beijing 100124, China; viola1341709539@163.com; 4 School of Mechanical Engineering & Automation, Beihang University, 37 Xueyuan Road, Haidian District, Beijing 100191, China; mycat123@gmail.com

**Keywords:** plasmonic nanostructure, superstructure, laser near-field reduction, SERS, PFOA

## Abstract

Surface-enhanced Raman scattering (SERS) enables trace-detection for biosensing and environmental monitoring. Optimized enhancement of SERS can be achieved when the energy of the localized surface plasmon resonance (LSPR) is close to the energy of the Raman excitation wavelength. The LSPR can be tuned using a plasmonic superstructure array with controlled periods. In this paper, we develop a new technique based on laser near-field reduction to fabricate a superstructure array, which provides distinct features in the formation of periodic structures with hollow nanoclusters and flexible control of the LSPR in fewer steps than current techniques. Fabrication involves irradiation of a continuous wave laser or femtosecond laser onto a monolayer of self-assembled silica microspheres to grow silver nanoparticles along the silica microsphere surfaces by laser near-field reduction. The LSPR of superstructure array can be flexibly tuned to match the Raman excitation wavelengths from the visible to the infrared regions using different diameters of silica microspheres. The unique nanostructure formed can contribute to an increase in the sensitivity of SERS sensing. The fabricated superstructure array thus offers superior characteristics for the quantitative analysis of fluorescent perfluorooctanoic acid with a wide detection range from 11 ppb to 400 ppm.

## 1. Introduction

Plasmonic structure arrays have recently gained much attention due to relevant applications such as sensing, photocatalysis, and light manipulation at the nanoscale. The important feature of plasmonic structure arrays for these applications is the tunability of the localized surface plasmon resonance (LSPR) from the visible to the infrared regions by adjustment of the period due to additional diffractive resonance [1,2]. Recently, Zhou et al. built up Ti nanorings on a conductive film for the solar energy absorber. Due to the plasmon resonance of Ti nanorings, the film has a broadband solar energy absorption, achieving a 97.0% absorption in a simulated ideal solar light source [3]. Herrmann et al. used femtosecond laser to thread gold nanoparticle strings for tuning surface plasmon. They verified the resonance mode depended on the nanoparticle size, the chain length and the peak laser power [4]. Yin et al. assembled hybrid Au-Ag nanoparticles and nanochains to couple the surface plasmon for the enhancement of absorption in the visible region [5]. Compared to the pristine nanoparticles, the hybrid Au-Ag nanochains displayed superior photocatalytic performances in hydrogenation under visible light irradiation.

Among the applications of LSPR, the tenability of LSPR can be applied to improve the performance of surface-enhanced Raman scattering (SERS) sensors because optimal LSPR tuned to match the Raman excitation wavelength can provide an additional increase in the electric field within each point [6]. The plasmonic structure arrays for SERS are commonly composed of highly ordered two-dimensional (2D) or 3D nanopoints, each of which consists of nanoparticles to modulate the light interaction in the nanometer-sized gaps. The relevant technique for the fabrication of plasmonic structure arrays is the template-assisted assembly of metal nanoparticles, reported by Odom’s [7], Fery’s [8] and Liz-Marzán’s [9] groups. Other methods, such as ion-beam lithography or photography, are time-consuming for large-area fabrication or do not have sufficient fabrication resolution to form the ordered nanoclusters. Although uniform plasmonic structure arrays have been efficiently obtained with high reproducibility using template-assembly, the fabrication procedure includes multiple steps such as template fabrication, nanoparticle synthesis, and transfer printing, which are complex, and the synthesis of nanoparticles is environmentally unfriendly due to the chemicals used.

Here, we develop a simple technique based on the laser near-field reduction of metal ions, which allows the production of plasmonic superstructure arrays with flexible periods with fewer steps and fewer chemicals than the present techniques. This technique uses silica microspheres that focus the laser beam at their bottom sides to induce the near-field. Reduction is confined in the near-field; therefore, silver nanoparticles are first grown at the bottoms of each microsphere and then the nanoparticles further grow along the silica microsphere surfaces to form silver nanoclusters. Self-assembled silica microspheres can form a highly ordered nanocluster array over a wide area. This technique provides a novel way to construct a plasmonic superstructure array composed of periodic nanostructures with hollow nanoclusters. The morphology of the nanoclusters can be controlled by irradiation with either a continuous wave (CW) laser or femtosecond laser, which is critical to the performance of SERS sensors. The plasmonic superstructure arrays fabricated are applied for wide-range analysis of perfluorooctanoic acid (PFOA) by taking advantage of the tunability in SERS analysis. PFOA is an artificial per- and polyfluoroalkyl substance that has been widely used in the last 50 years for industrial applications such as textiles and sealants. However, it can damage health if ingested from drinking water or food [10]. In recent years, the use of PFOA and similar chemicals has been prohibited worldwide. However, trace levels of PFOA can still be found in the environment. The problem of sensing PFOA by SERS is that it is a strongly fluorescent material, so that the fluorescence induced by the Raman excitation laser may conceal Raman signals. Longer Raman excitation wavelengths, e.g., 633 nm, can suppress the fluorescence; however, this can also reduce the Raman intensity because of the lower photon energy when using common silver nanoparticles. Therefore, silver plasmonic superstructure arrays with LSPR tuned for the longer wavelength by laser near-field reduction are applied for the wide-range analysis of PFOA.

## 2. Materials and Methods

**Figure 1:**
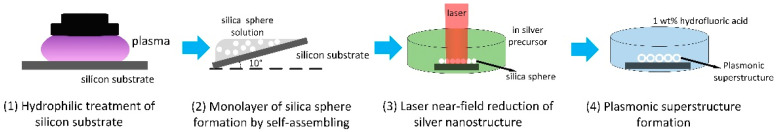
The schematic illustration for fabrication procedure of plasmonic superstructure, which includes.

The fabrication of plasmonic superstructure consists of four steps as shown in Figure 1: (1) Silicon substrate was treated by plasma soft etcher to decrease the contact angle of a solution containing silica spheres; (2) The silica sphere solution was dropped on 10° tilted silicon substrate to form a monolayer of silica sphere due to self-assembling at room temperature; (3) silver nanoclusters were synthesized on the self-assembled silica spheres by laser near-field reduction in the pre-prepared silver precursor; (4) The laser-treated sample was washed by 1 wt% hydrofluoric acid solution to eliminate silica spheres.

### 2.1. Silver Precursor Preparation

0.08 M silver nitrate and 0.06 M trisodium citrate were mixed to form a white suspension in 10 mL of pure water, then 0.3 mL ammonia solution (28 wt%) was added to the suspension to obtain a transparent solution.

### 2.2. Monolayer Formation of Silica Microspheres

A monolayer of silica microspheres was produced on a silicon substrate by the self-assembly method. Briefly, a 2-inch silicon wafer was cut into 10 mm × 10 mm pieces and 2 wt% silica microsphere solution diluted with ethanol was dropped onto the silicon substrate tilted at 10° at room temperature. The solution withdrew slowly from the top of the substrate due to evaporation, so that silica microspheres were gradually self-assembled and formed a close-packed monolayer. Silica microspheres with diameters of 500, 1000, and 2000 nm were used in these experiments.

### 2.3. Laser Near-Field Reduction

Figure S1 in Supplementary Material shows a schematic illustration of the experimental setup for laser near-field reduction. The laser source used was a commercially available 405 nm CW laser (Cobolt 06-01, Hübner, Germany), or a 1030 or 515 nm femtosecond laser with a pulse width of 223 fs and a repetition rate of 100 kHz (Carbide, Light Conversion, Vilnius, Lithuania). The silicon substrate covered with silica microspheres was immersed in a silver precursor solution and was immediately irradiated by either of the lasers. The laser beam was loosely focused or expanded by a lens with a focal length of 100 mm to achieve a 3 mm diameter beam spot. After laser near-field reduction, the silica microspheres were eliminated by (1 wt%) hydrofluoric acid solution to create hollow nanoclusters. The details of laser near-field reduction are given in the Supplementary Material.

### 2.4. Simulation of Local Electric Field on Plasmonic Superstructure

The local electric field induced on plasmonic superstructure was simulated by the method of finite element analysis. The model for simulation is described in Supplementary Material, Figure S2. Briefly, the periodic boundary conditions are used to account for the periodic nature of plasmonic superstructure. The incident illumination is a plane wave, introduced from Port 1 to Port 2. The superstructure array is surrounded by air. More detail of simulation can be found in the Supplementary Material.

### 2.5. Characterization and SERS Measurements

High-resolution SEM images and EDS analyses of the silver nanoclusters were obtained using an environmental SEM (Quattro, ThermoFisher, Waltham, MA, USA). Extinction spectra of plasmonic superstructure arrays were measured using a UV-Vis-NIR spectrophotometer (UV-3600i plus, Shimadzu, Japan). All Raman data were acquired at room temperature using a Raman spectrometer equipped with 633 and 785 nm excitation lasers (NRS-4500, Jasco, Japan) and two gratings (1800 grooves per mm for 633 nm and 1200 grooves per mm for 785 nm). A slit (50 × 8000 μm) was adopted to achieve a resolution of 2.3 cm^−1^. Before measurements, the spectrometer was calibrated using a standard silicon substrate. The excitation power and exposure time were set at 2.8 mW and 10 s, respectively, for measurement of Rhodamine 6G solution with 2 accumulations. Meanwhile, excitation power and exposure time were set at 1.4 mW and 1 s, respectively, for measurement of crystal violet mixed with PFOA solution with 2 accumulations. The Raman excitation laser was focused using a 50× objective lens with a numerical aperture of 0.5. Each sample was measured 5 times at the same conditions to acquire an averaged spectrum. For data treatment, the baseline function and the peak find function were used to delete the invalid data and to identify Raman peaks, respectively.

## 3. Results and Discussion

**Figure 2:**
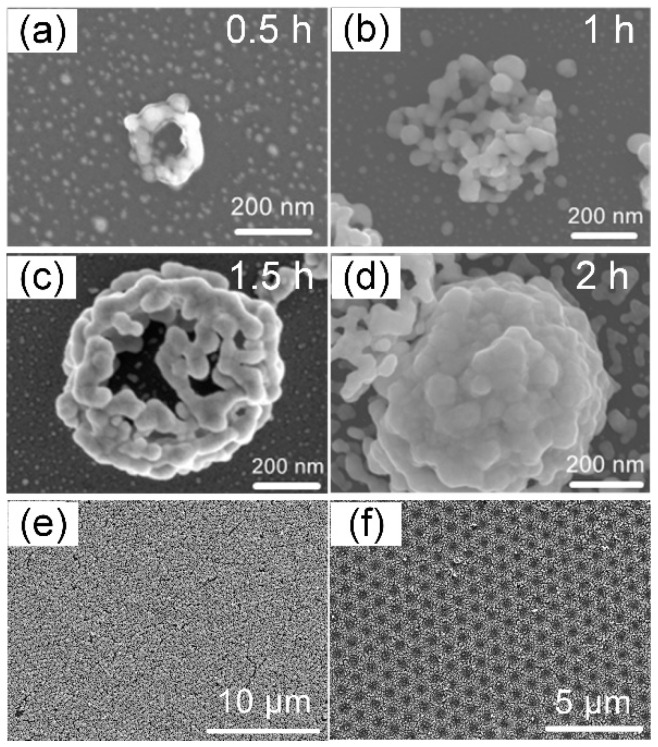
Observation of silver nanoclusters fabricated by laser near-field reduction. (**a**–**d**) SEM images of single silver nanocluster fabricated by CW laser near-field reduction with reduction times of 0.5, 1, 1.5, and 2 h after silica microsphere elimination. (**e**) Low magnification SEM image of plasmonic hollow nanocluster array with a period of 500 nm. (**f**) Crater array formation on a silicon substrate due to silica microspheres shielding against silver nanoparticle deposition.

Based on Mie theory, when the incident light wavelength is slightly longer than the diameter of a homogenous single dielectric sphere, a tightly focused beam with a subwavelength waist is generated on the shadow of the sphere [11]. Therefore, when the laser is irradiated onto silica microspheres, the laser beam can be focused by the microspheres to increase the electric field in a near-field distance on the bottom of the microspheres. Supplementary Material, Figure S4 shows a simulation of the laser propagation through a single silica microsphere with various diameters for irradiation with different laser wavelengths. The higher amplitude of the electric field in the laser focused area means that the silver ions have a greater probability of being reduced to form silver nanoparticles below the silica microspheres in the precursor than other laser paths. Figure 2a shows a ring structure with an outer diameter of 180 nm that was generated by irradiation of a 500 nm silica microsphere with a 405 nm CW laser at 0.7 mW/mm^2^ for 0.5 h. It is understandable that the formation of the ring structure results from the silver nanoparticles surrounding the contact location of the silica microsphere and the silicon substrate. The small silver nanoparticles that form the ring structures act as electron storage to transfer the electrons to the precursor medium for the subsequent reduction of silver ions [12]. Therefore, as the reduction time is increased, the silver nanoparticles grow along the surface of the silica microsphere to form the ring structure, becoming a nanocluster after 1 h or longer (Figure 2b,c and Supplementary Material, Figure S5a (cross-sectional scanning electron microscopy (SEM) image)), and completely wrapping the silica microsphere after 2 h (Figure 2d). The reduction time for complete wrapping is dependent on the laser intensity; therefore, control of the laser intensity and reduction time can create bowl nanostructures (see Supplementary Material, Figure S5b) or hollow nanoclusters (see Supplementary Material, Figure S5c) after elimination of the silica spheres using hydrofluoric acid. Such bowl and hollow nanoclusters have gained attention in recent years due to physical properties such as high specific surface area, and structural properties that benefit the applications for plasmonic devices and energy storage [13,14,15]. Although the hollow nanoclusters are generally synthesized by wet-chemical methods, the laser near-field reduction can create pure metal nanostructures because it does not require additional agents such as photoinitiators and surfactants. The laser near-field reduction using the self-assembled silica microspheres presented here can be employed to fabricate an array of hollow nanoclusters with adjustable periods, which cannot be achieved by wet-chemical methods [16,17]. Figure 2e shows a nanocluster array with a period of 500 nm fabricated by CW laser near-field reduction. In this experiment, the laser spot was 7 mm^2^, which enabled the formation of plasmonic superstructure arrays over an area larger than the requirements for SERS applications. Removal of the silica microspheres results in a crater array where the silver nanoparticles are not deposited, because the silica microspheres shield the silicon substrate from deposition of the silver nanoparticles (Figure 2f).

**Figure 3:**
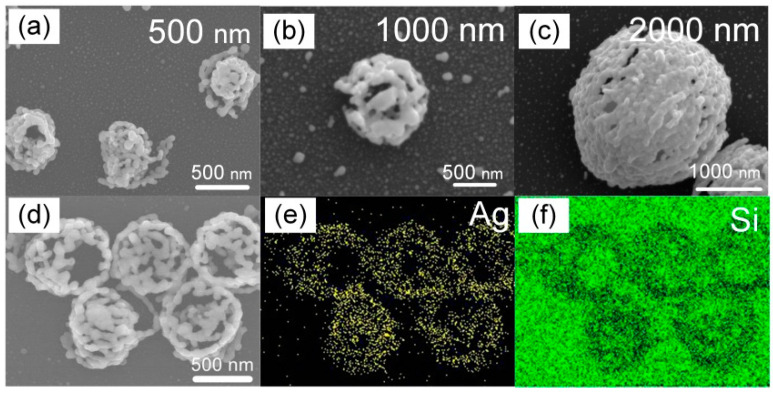
Morphology of silver nanocluster with different sizes. Silver nanocluster with sizes of (**a**) 500 nm, (**b**) 1000 nm, and (**c**) 2000 nm. (**d**) Magnified SEM image of five silver nanoclusters with a size of 500 nm, and corresponding EDS maps for (**e**) silver and (**f**) silicon.

**Figure 4:**
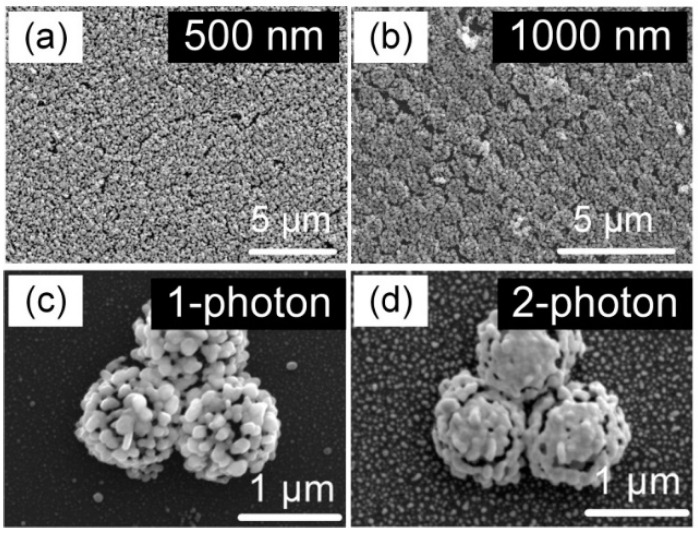
Single-photon absorption and two-photon absorption induced reduction for fabrication of plasmonic hollow nanocluster arrays. Plasmonic hollow nanocluster arrays with periods of (**a**) 500 nm and (**b**) 1000 nm fabricated using a CW laser. High magnification SEM images of 1000 nm period nanoclusters fabricated by laser near-field reduction based on (**c**) single-photon absorption and (**d**) two-photon absorption.

Silver nanoparticles grow along the surfaces of the silica microspheres; therefore, the period of the nanocluster array can be easily adjusted using different diameters of silica microspheres. For example, Figure 3a,b show nanoclusters formed on loosely assembled silicon microspheres with diameters of 500 nm and 1000 nm, respectively, where the sizes of the nanoclusters coincide with those of the silicon microspheres. Different periods of plasmonic superstructure arrays can thus be fabricated, as shown in the Figure 4a,b and Supplementary Material, Figure S6. The energy dispersive X-ray spectroscopy (EDS) mapping images shown in Figure 3d,e, and the corresponding SEM image (Figure 3d) indicate that the nanoclusters are composed of pure silver.

**Figure 5:**
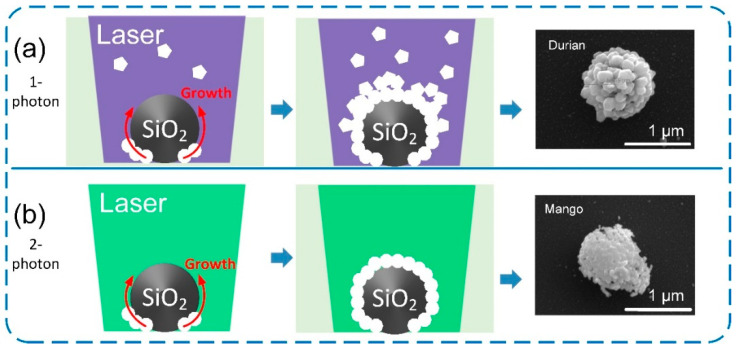
Growth mechanisms of plasmonic hollow nanocluster arrays. Growth mechanisms of plasmonic hollow nanocluster arrays fabricated by laser near-field reduction based on (**a**) single-photon and (**b**) two-photon absorption.

Laser near-field reduction for the formation of a silver nanocluster array with a femtosecond laser was also demonstrated to investigate whether single or multiphoton photoreduction is effective at achieving plasmonic superstructure arrays with higher performance. Figure 4a,b show silver nanocluster arrays with 500 nm and 1000 nm periods fabricated by CW laser near-field reduction with a wavelength of 405 nm. Both images show the silver nanoclusters generated by the reduction are closely packed and highly ordered. For comparison, 1000 nm and 2000 nm periods of nanocluster arrays were fabricated by femtosecond laser with wavelengths of 515 nm and 1030 nm, respectively, as shown in Supplementary Material, Figure S6. The silver precursor has an absorption peak at 302 nm (see Supplementary Material, Figure S7); therefore, the silver ion reduction induced by both 515 nm and 1030 nm femtosecond lasers can be attributed to multiphoton absorption [18]. This multiphoton reduction leads to silver nanoclusters with different morphology from those formed by single-photon reduction. Figure 4c,d compare three adjacent silver nanoclusters generated by single-photon reduction and two-photon reduction, respectively. For single-photon reduction, the silver nanoparticles that form the nanocluster show a clear boundary, whereas for two-photon reduction, the silver nanoparticles are fused to each other due to localized melting induced by the high intensity of the femtosecond laser [19], which results in an unrecognizable boundary. On the other hand, additional silver nanoparticles are deposited on the nanoparticles covering the silica microspheres by single photon absorption. Single-photon reduction generates additional silver nanoparticles in the silver precursor solution, although production is much lower than that in the laser near-field. Once the silver nanoparticles are generated on the silicon nanospheres, these silver nanoparticles enhance the electric field because of LSPR induced by the 405 nm CW laser, which promotes the further growth of silver nanoparticles. The additional nanoparticles are deposited on the nanoclusters, which results in distinguishable boundaries. In contrast, the absorption cross-section for *n*-photon absorption is proportional to the *n*-th power of the laser intensity; therefore, the laser reduction can be confined only to the laser focused region to suppress photoreduction in the precursor [20,21]. The longer wavelength of the femtosecond laser provides little possibility of inducing LSPR on the silver nanoparticles. Photoreduction mechanisms for each scheme are illustrated in Figure 5. A durian-like nanocluster (Figure 5a) is eventually formed by single-photon absorption due to the additional deposition of silver nanoparticles from the precursor, while a mango-like nanocluster (Figure 5b) is formed by the multiphoton reduction because it confines the reduction to the near-field and then induces localized fusing of nanoparticles formed on the surface silica microspheres.

**Figure 6:**
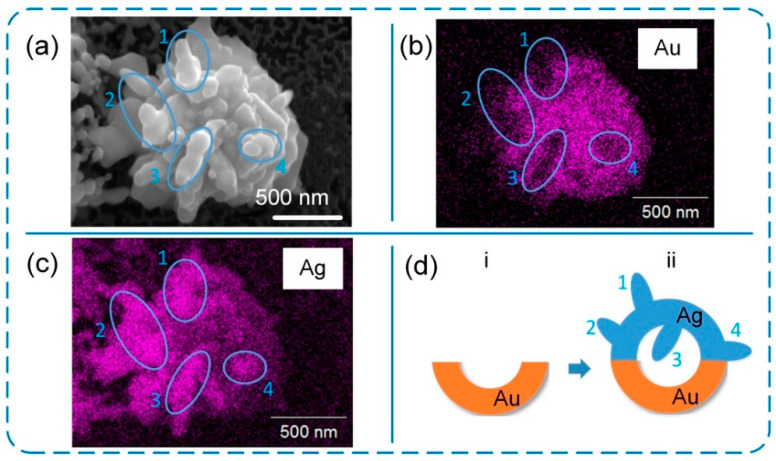
Silver/gold hybrid nanostructure. (**a**–**c**) SEM image and corresponding EDS mappings of Au and Ag. (**d**) Schematic illustration of single Ag/Au hollow nanocluster.

The laser near-field reduction can also be used to fabricate plasmonic superstructure arrays using gold. As shown in Supplementary Material, Figure S8, a gold plasmonic superstructure array with a period of 1 μm is formed with a highly ordered nanostructure, which demonstrates the versatility of the present method. This highly ordered structure generates a colorful surface (see insert in Supplementary Material, Figure S8a) due to light diffraction, which demonstrates the intrinsic properties of plasmonic superstructure array. Furthermore, a hybrid superstructure of gold/silver microspheres can be fabricated by laser near-field reduction. For example, we successfully fabricated a hybrid microsphere composed of a gold bottom hemisphere covered with a silver top hemisphere using a two-step process, as shown in Figure 6. The long reduction time (2 h) and high power density (1.5 mW/mm^2^) of the 405 nm CW laser were applied to reduce silver ions, so that protruding nanostructures were formed on the top of the microsphere, which are labeled as regions No. 1–4. Based on the EDS results, regions No. 1–4 are silver nanostructures formed on the silver top hemisphere (Figure 6c), which obscures the signals in gold EDS mapping (Figure 6b). The silver and gold hemispheres complementarily constitute an entire microsphere to build up a hybrid structure as illustrated in Figure 6d.

**Figure 7:**
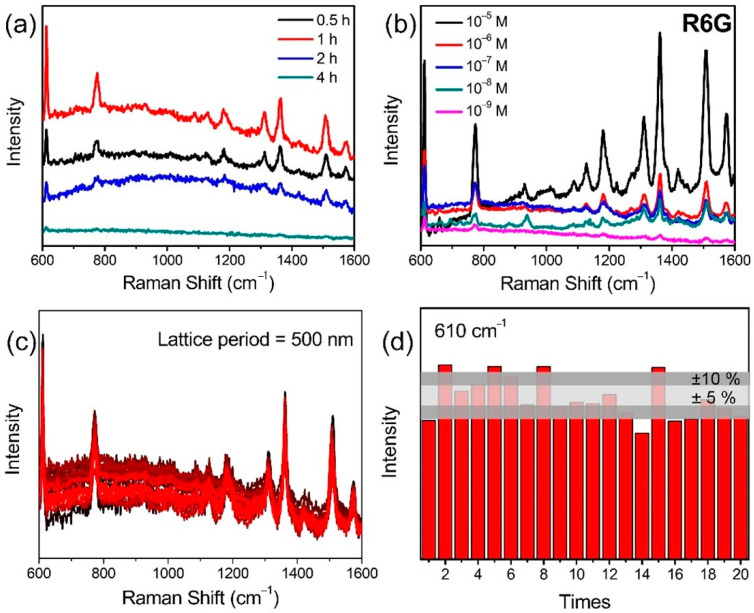
SERS performance of plasmonic hollow nanocluster arrays. (**a**) SERS spectra for R6G (10^−6^ M) on plasmonic hollow nanocluster arrays with various reduction times. (**b**) SERS spectra for various concentrations of R6G on plasmonic hollow nanocluster arrays fabricated with a 405 nm CW laser at a power density of 1.4 mW/mm^2^. The period of the plasmonic hollow nanocluster arrays was 500 nm. (**c**) SERS results of 10^−6^ M R6G solution collected from 20 randomly selected locations on a hollow nanocluster array. (**d**) Fluctuations of SERS results for 10^−7^ M R6G solution at 610 cm^−1^.

Prior to the PFOA analysis, the SERS performance of plasmonic superstructure arrays constructed of an array of silver nanoclusters was evaluated using R6G molecules. We measured the SERS spectra of plasmonic superstructure arrays with a period of 500 nm, fabricated with a CW laser for different reduction times from 0.5 to 4 h. Figure 7a shows that the Raman intensity increased with the reduction time, and the maximum was obtained for a reduction time of 1 h, which then decreased at 2 h and longer times. For a reduction time of 1 h, small gaps between each nanoparticle in the silver nanoclusters were formed to induce the plasmonic mode, which provided the highest SERS intensity due to the optimal amount of nanoparticles in the nanocluster [22]. However, as the reduction time was increased, the gaps gradually filled and nanoparticles were misordered due to excess reduction. The plasmonic mode for such a structure can no longer be effectively excited, which leads to deterioration of the SERS capacity [23]. The analytical enhancement factor (AEF) was evaluated for the plasmonic superstructure array obtained with a reduction time of 1 h, where the maximum Raman intensity was obtained. Figure 7b shows the Raman signals for different concentrations of R6G induced by an excitation laser with a wavelength of 633 nm, which were collected to calculate the AEF using the following equation: AEF = (*I*_SERS_/*I*_OR_)/(*C*_SERS_/*C*_OR_), where *I*_SERS_ and *C*_SERS_, and *I*_OR_ and *C*_OR_ correspond to the Raman intensities of R6G and the molar concentrations of the R6G solution on the SERS substrate and on the silicon wafer, respectively [24]. The AEF of R6G at 612 cm^−1^ was estimated to be 2.3 × 10^7^ with the detection limit determined to be as low as 10^−9^ M. The detection limit (signal/noise = 3/1) was defined as the lowest concentration that can be detected by spectrometer [25,26,27]. In addition, the SERS performance of the plasmonic superstructure arrays fabricated by single- and two-photo absorption were compared using R6G molecules at concentrations of 10^−7^ M and 10^−8^ M, which is shown in Supplementary Material, Figure S9. As shown in Figure 4d, the two-photon absorption allows the nanogaps between silver nanoparticles in the nanocluster to vanish due to femtosecond laser-induced melting. Therefore, the electric field surrounding the nanocluster cannot be effectively enhanced to excite the plasmonic mode, which leads to inferior performance compared to the single-photon reduction sample. It was thus concluded that single-photon reduction using the CW laser is suitable for the fabrication of plasmonic superstructure arrays due to the resultant hollow nanostructure, which is an array of hollow nanoclusters composed of nanoparticles. The reproducibility is also an important aspect to evaluate the SERS performance [25]. SERS results of 10^−6^ M R6G solution were collected at 20 randomly selected locations in the 50 × 50 μm area of the plasmonic superstructure array. The relative standard deviation (RSD) of the plasmonic superstructure array is approximately 9.6% (Figure 7c), which is relatively higher than the previous work (6%) [6], while the analysis can be completed in a very short time (within a minute) in our experiments due to the lack of an incubation process (1 h for incubation in Ref. [6]).

**Figure 8:**
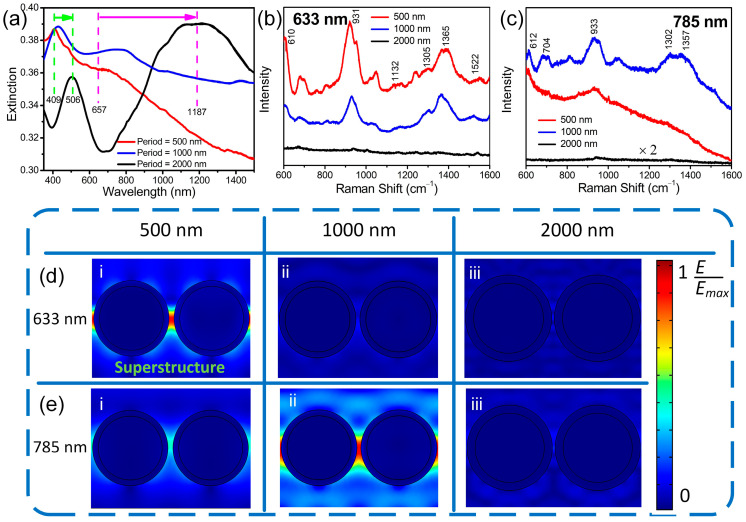
Tunability of plasmonic superstructure array. (**a**) Extinction spectra obtained from plasmonic superstructure arrays with periods of 500, 1000, and 2000 nm. (**b**,**c**) SERS spectra obtained from R6G (10^−7^ M) on the plasmonic superstructure arrays with periods of 500, 1000, and 2000 nm using (**b**) 633 and (**c**) 785 nm Raman excitation lasers. (**d**,**e**) Electric field distributions induced by silver nanoclusters with periods of (**i**) 500, (**ii**) 1000, and (**iii**) 2000 excited at (**d**) 633 and (**e**) 785 nm. The color bar on the right side indicates the electric field E normalized by the maximum enhanced electric field E_max_ (E/E_max_).

The LSPRs of the plasmonic superstructure arrays fabricated with the CW laser were then measured using a spectrophotometer. Figure 8a shows the extinction spectra of the plasmonic superstructure arrays with periods of 500, 1000, and 2000 nm. Two LSPRs are observed in the extinction spectra, which are red-shifted with an increase in the period. The first LSPR arises from the dipole resonance of the silver nanoclusters, which is red-shifted from 409 to 506 nm because of the increase in the silver nanocluster size. The second LSPR is a plasmonic mode that can be tuned from 657 nm to 1187 nm by adjustment of the period. The SERS intensity obtained by the 500 nm period with the 633 nm Raman excitation wavelength is higher than 1000 and 2000 nm periods due to the tunable plasmonic mode mode, resulting from the enhancement of LSPR, particularly at 610 cm^−1^. In fact, in Figure 8b, the peak height at 610 cm^−1^ for the 500 nm period (red curve) is significantly higher than the 1000 and 2000 nm periods (blue and black curves, respectively). The SERS intensity for the 1000 nm period at a Raman excitation wavelength of 785 nm is higher than the others (Figure 8c). In Figure 8d,e, the tunability of the plasmonic superstructure arrays is demonstrated by simulating the spatial distribution of the electric field, for which detailed information, such as the nanoparticle sizes and model used for the simulation, is given in the Supplementary Material, Figures S2 and S3. To simplify the simulation, a pair of hollow silver nanoclusters was used with a periodic boundary condition to simulate the electric field enhanced by the superstructure array, where the gap between the two silver nanoclusters was set as 34 nm for 500 nm period, 54 nm for 1000 nm and 64 nm for 2000 nm. All dimensions of the model for simulation were determined from SEM images. When the 633 nm light is incident, the 500 nm period generates the highest electric field amplitude in the hotspot, which is 6.5 and 8.2 times higher than that excited by the 1000 and 2000 nm periods, respectively. On the other hand, in the case of 785 nm incident light, the 1000 nm period achieves 2.1- and 5.5-times higher amplitudes than the 500 and 2000 nm periods, respectively. Therefore, it is reasonably concluded that the laser near-field reduction presented here can realize the formation of tunable plasmonic superstructure arrays.

**Figure 9:**
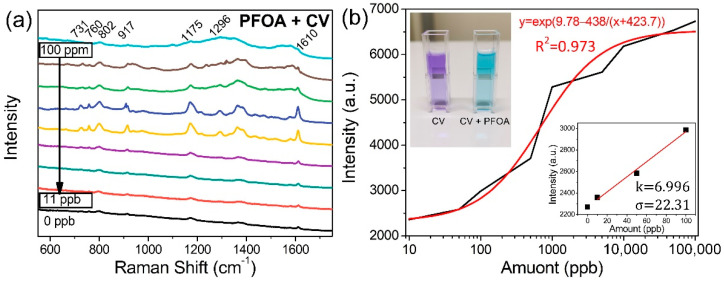
SERS analysis of PFOA using plasmonic hollow nanocluster array. (**a**) SERS spectra of 10^−7^ M CV mixed with different concentrations of PFOA, measured using the plasmonic superstructure array with a period of 500 nm. (**b**) The calibration curve for the sensing of PFOA for the Raman peak at 1175 cm^−1^ (inset: photograph of CV solution (**left**) and CV mixed with PFOA solution at a concentration of 100 ppm (**right**)). The Raman excitation wavelength was 633 nm.

In the final part, the plasmonic superstructure arrays were applied for the quantitative analysis of PFOA. Although PFOA is valuable for industrial products, it can persist in the environment, which makes it a high health risk if ingested from drinking water or food [10]. The bioaccumulation of PFOA over time may influence the immune system and potentially cause cancer. PFOA produces strong fluorescence during Raman measurement (as illustrated in Supplementary Material, Figure S10). The 500 nm period plasmonic superstructure array fabricated with the CW laser was used due to its LSPR at 657 nm. Although the laser near-field reduction technique can create a hybrid superstructure of gold/silver microspheres, LSPR of the hybrid structure should be longer than 657 nm due to the longer LSPR of gold. Therefore, the silver plasmonic superstructure was used for this measurement, because its LSPR at 657 nm is much closer to Raman excitation wavelength (633 nm) used. PFOA was mixed with CV rather than attempting direct detection. Addition of CV can significantly lower the power of Raman excitation laser and shorten the exposure time as compared with direct detection of PFOA, which is beneficial to suppress fluorescence from PFOA for accurate sensing. Supplementary Material, Figure S11 shows that 633 nm is out of the absorption peak for CV mixed with PFOA, so that the excitation of strong fluorescence could be avoided. Loading the PFOA into CV molecules enabled the CV to exhibit higher Raman activity due to the formation of ion-pairs between CV and PFOA. These ion-pairs are beneficial for Raman measurements because they strengthen the Raman activity by the increase in the amount of analyte molecules loaded into the plasmonic superstructure arrays. The formation of these ion-pairs did not shift the Raman scattering wavenumber of CV [28]. In Figure 9a, the 10^−7^ M pure CV solution was used as a baseline. The Raman intensity increases with the amount of PFOA due to the increased amount of ion-pairs. The lower detection limit of PFOA can be defined as 3.3 σ/k, where σ is the standard error of the y-intercept in the regression line and k is the slope according to IUPAC definition [29,30]. Since this definition relies on a linear relationship, the lower detection limit is calculated in the lower concentration range of 0–100 ppb, which is 10.52 ppb. When the concentration of PFOA reaches 100 ppm, however, two broad peaks associated with the fluorescence of PFOA are observed in the range of 1000–1620 cm^−1^, which overlap with the Raman signals, so that they cannot be detected. Therefore, this technique cannot be used to detect PFOA with concentrations higher than 100 ppm which is defined as the upper detection limit. Figure 9b shows a calibration curve of the Raman intensity of CV at 1175 cm^−1^ for different concentrations of PFOA, which almost approximates an exponential increase in the Raman intensity as a function of the PFOA concentrations in the range of 11 ppb to 100 ppm with y = exp(9.78 − 438/(x + 423.7)), where x is the concentration of PFOA and y is the Raman intensity of CV. The exponential relationship between the PFOA and Raman intensity may be attributed to the enhancement of Raman scattering based on SERS, since the enhancement non-linearly increases as the analyte concentration increases [31,32,33,34]. A larger period of the plasmonic superstructure array enables the detection of higher concentrations of PFOA because the LSPR matches the longer Raman excitation wavelength, although the detection limit is sacrificed. For example, as shown in Supplementary Material, Figure S12, the detection range of a plasmonic superstructure array with a period of 1000 nm, of which the LSPR matches the 785 nm Raman excitation wavelength, is from 50 ppb to 400 ppm. The upper detection limit is determined by the fluorescence intensity of PFOA, since the fluorescence of PFOA with high concentration veils the Raman peaks of CV. Based on the simulation and experimental results, the 1000 nm periodic superstructure achieves higher enhancement of electric field than the 500 nm periodic superstructure at the excitation wavelength of 785 nm. Additionally, the electric field enhanced by the 1000 nm periodic superstructure with 785 nm wavelength is lower than that by the 500 nm period with 633 nm wavelength. The lower electric field as well as the longer excitation wavelength are beneficial to suppress fluorescence, while the enhanced electric field is still high enough to detect PFOA with concentration down to 50 ppb. Therefore, the 1000 nm periodic superstructure gives a higher upper detection limit than the 500 nm periodic superstructure. It should be noted that the combined use of plasmonic superstructure arrays with different periods enable the coverage of a wider detection range, and the laser near-field reduction can be used to easily and precisely adjust the periods. Additionally, we evaluated the ability of PFOA detection when mixing another polymeric material (polyvinylpyrrolidone (PVP)). Figure S13 shows that the PVP has little influence on the intensity of Raman signal since only PFOA can increase the peak height of CV due to the formation of ion-pairs. Therefore, the PFOA can be selectively detected by the indirect measurement using plasmonic superstructure array.

Table 1 compares the performance of several methods for PFOA detection. Although mass spectrometry has much lower detection limits [35,36,37,38], it is time-consuming and highly costly due to the requirement of pretreatments. Mass spectrometry also has a very narrow detection range, so that concentrations higher than 50 ppb cannot be analyzed. In contrast, the fluorescence method is convenient and simple because it only requires a fluorescence microscope and is capable of detecting relatively higher concentrations [39,40,41,42,43]. Conventional SERS using silver nanoparticles achieves detection limits similar to the fluorescence and light scattering methods, although its detection range is much narrower [22]. Most importantly, SERS that uses the plasmonic superstructure array with a hollow nanocluster presented in this work has a much wider quantitative detection range from 11 ppb to 400 ppm with a similar detection limit. Therefore, considering the advisory level to avoid damage to health (70 ppb by the United States Environmental Protection Agency; 50 ppb by the Japanese Ministry of the Environment) in drinking water, the technique presented here is suitable as a general method to quantitatively analyze PFOA with a wide range of concentrations, and provides advantages of time saving and cost-effectiveness to monitor PFOA in the environment [44,45].

## 4. Conclusions

Plasmonic superstructure arrays composed of periodic structures with hollow nanoclusters were fabricated by the laser near-field reduction of silver ions, which showed the capacity of flexibly tuning the LSPR in the visible range to the near-infrared region. Silica microspheres self-assembled on silicon substrates focused the laser beam at their rear sides to generate optical near-fields with enhanced electric fields. Elimination of the silica microspheres using hydrofluoric acid solution after the entire growth process left plasmonic superstructure arrays with hollow nanoclusters on the silicon substrates with periods of 500 to 2000 nm, which were governed by the diameters of the silica microspheres. The plasmonic superstructure arrays fabricated by the single-photon absorption were superior in terms of enhancement of the SERS intensity due to the hollow nanocluster associated with the nonthermal nature of this process compared with those fabricated by multiphoton absorption using a femtosecond laser. R6G molecules were applied to evaluate the tunability of the plasmonic superstructure arrays and demonstrated that the LSPR could be tuned so as to match the Raman excitation wavelengths. The RSD of the plasmonic superstructure arrays was evaluated to be 9.6% due to the periodic nanocluster. The area where plasmonic superstructure arrays can be formed at once is determined by the laser spot size, and the fabrication of plasmonic superstructure arrays with centimeter areas is available by expanding the laser spot. Plasmonic superstructure arrays with hybrid Ag/Au nanostructures were fabricated to demonstrate that the developed technique provides a novel pathway to create an innovated nanostructure. Such unique plasmonic superstructure arrays enabled the quantitative detection of PFOA by the collaboration of CV solution with an exponential relationship in the range of 11 ppb to 400 ppm and a detection limit of 11 ppb. The technique presented in this work represents a novel, time-saving, cost-effective method for the trace analysis of fluorescent materials such as PFOA and other SERS-related applications.

## Figures and Tables

**Table 1:** Performance of each method for the detection of trace PFOA.

Methods	Quantitative Detection Range (ppb)	Detection Limit (ppb)	Ref.
Mass spectrometry	NA	0.011	[35]
Mass spectrometry	0.1–50	NA	[36]
Mass spectrometry	0.103–0.223	NA	[37]
Mass spectrometry	0.05–1	0.025	[38]
Fluorescence	NA	10	[39]
Fluorescence	100–6000	10	[40]
Fluorescence	4000–28,000	720	[41]
Fluorescence	20–4000	5	[42]
Fluorescence	41–41,000	NA	[43]
Light scattering	40–10,000	4.4	[46]
SERS	50–500	50	[21]
SERS	11–100,000 (500 nm period) 11–400,000 (combined use of 500 nm and 1000 nm periods)	11	Present work

## Data Availability

Not applicable.

## Supplementary Materials

The following supporting information can be downloaded at: https://www.mdpi.com/article/10.3390/nano12060970/s1, Figure S1: Schematic illustration of laser near-field reduction for the fabrication of plasmonic superstructure arrays; Figure S2: Model of plasmonic superstructure array for the simulation of electric fields around the nanocluster; Figure S3: Typical SEM images and size distributions of nanoparticles; Figure S4: The propagation of laser through the silica microsphere; Figure S5: SEM images of the nanocluster from cross sectional view, bowl and hollow nanoclusters; Figure S6: Plasmonic superstructure arrays with periods of 1000 nm and 2000 nm; Figure S7: Absorption spectrum of silver precursor solution; Figure S8: Large area image of gold plasmonic superstructure array and single gold nanocluster; Figure S9: SERS intensities of R6G molecules using the plasmonic superstructure arrays fabricated by singe- and two-photon absorption; Figure S10: Raman spectrum of 4 ppm PFOA in pure water on superstructure array; Figure S11: The absorbance of crystal violet solution mixed with PFOA; Figure S12: SERS spectra of the crystal violet mixed with PFOA using 1000 nm the plasmonic superstructure arrays; Figure S13: A comparison of CV solution mixed with polyvinylpyrrolidone and perfluorooctanoic acid. Reference [47] is cited in the Supplementary Materials.
