# Supplementary material for: Plasmonic Superstructure Arrays Fabricated by Laser Near-Field Reduction for Wide-Range SERS Analysis of Fluorescent Materials
Source: Nanomaterials (Basel). 2022 Mar 15;12(6):970. doi: 10.3390/nano12060970 (PMC8950659; doi:10.3390/nano12060970)
Supplement: Supplementary file 1 [file nanomaterials-12-00970-s001.zip › nanomaterials-1624329-supplementary.pdf]

nanomaterials

MDPI

Supplementary Materials

Plasmonic Superstructure Arrays Fabricated by Laser Near-Field Reduction for Wide-Range SERS Analysis of Fluorescent Materials

Shi Bai [1], Anming Hu [2], Youjin Hu [3], Ying Ma [4], Kotaro Obata [1] and Koji Sugioka [1,*]

[1] Advanced Laser Processing Research Team, RIKEN Center for Advanced Photonics, 2-1 Hirosawa, Wako, Saitama 351-0198, Japan; shi.bai@riken.jp (S.B.); kobata@riken.jp (K.O.)
[2] Department of Mechanical, Aerospace and Biomedical Engineering, University of Tennessee Knoxville, 1512 Middle Drive, Knoxville, TN 37996, USA; ahu3@utk.edu
[3] Institute of Laser Engineering, Faculty of Materials and Manufacturing, Beijing University of Technology, 100 Pingle Yuan, Beijing 100124, China; viola1341709539@163.com
[4] School of Mechanical Engineering & Automation, Beihang University, 37 Xueyuan Road, Haidian District, Beijing 100191, China; mycat123@gmail.com
* Correspondence: ksugioka@riken.jp

Experimental

Material. Ammonia solution (guaranteed reagent) (Junsei Chemical Co. Ltd, Tokyo, Japan) and trisodium citrate dihydrate (guaranteed reagent) (Junsei Chemical Co. Ltd, Tokyo, Japan) were purchased. Silver nitrate (guaranteed reagent) (Fujifilm Wako Pure Chemical Corporation, Osaka, Japan) and hydrofluoric acid solution (guaranteed reagent) (Fujifilm Wako Pure Chemical Corporation, Osaka, Japan) were purchased.

Hydrophilic treatment of silicon wafer. 2 inches N-type (12.8–15.7 Ω•cm) silicon wafer was treated by a plasma soft etcher (PIB-20, Vacuum Device, Mito, Japan) for 1 min to increase the hydrophilicity. The plasma was created in atmosphere with a pressure of 20 Pa and a discharge current of 20 mA. Then, the silicon wafer was cut into 10 mm × 10 mm pieces as the silicon substrate.

Laser near-field reduction. 1.5 ml silver precursor was poured into a cylindrical vessel, and then the silica microspheres coated silicon wafer was immersed into the precursor. The lasers were focused or expanded to achieve 3 mm spot size on silicon wafer at 0.7 mW/mm^2 of continuous wave laser (405 nm) and 10 nJ/mm^2 of femtosecond laser (1030 nm, 515 nm). The repetition rate of femtosecond laser was 100 kHz and pulse during time was 223 fs. The second harmonic (515 nm) of femtosecond laser was generated using a lithium triborate (LBO) single crystals.

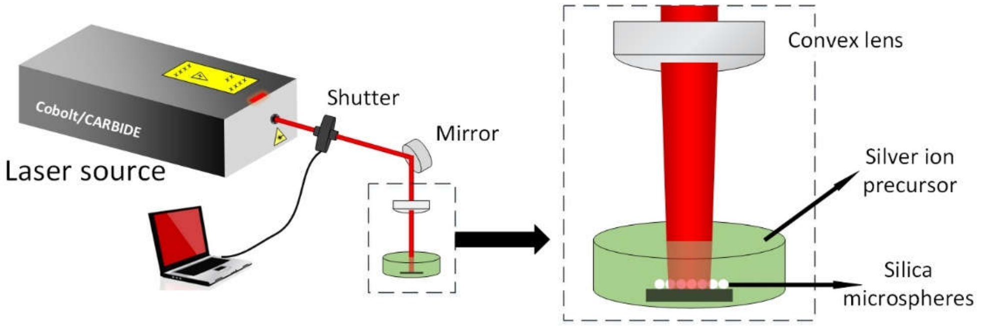

Figure S1. Schematic illustration of laser near-field reduction for the fabrication of plasmonic superstructure arrays.

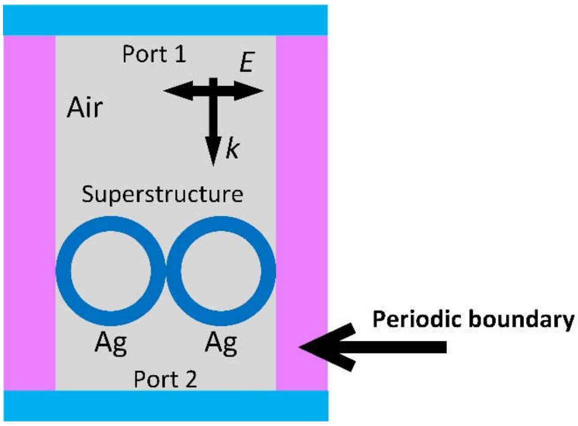

Figure S2. Model of plasmonic superstructure arrays for the simulation of electric fields around the nanocluster. The geometry of superstructure is determined from SEM images shown in Figure S3. The plane wave is incident from the top port and the boundaries are set at periodic boundaries to simulate the electric field enhanced by a pair of silver Ag spherical hollow structures. The material properties are based on the Ref [1].

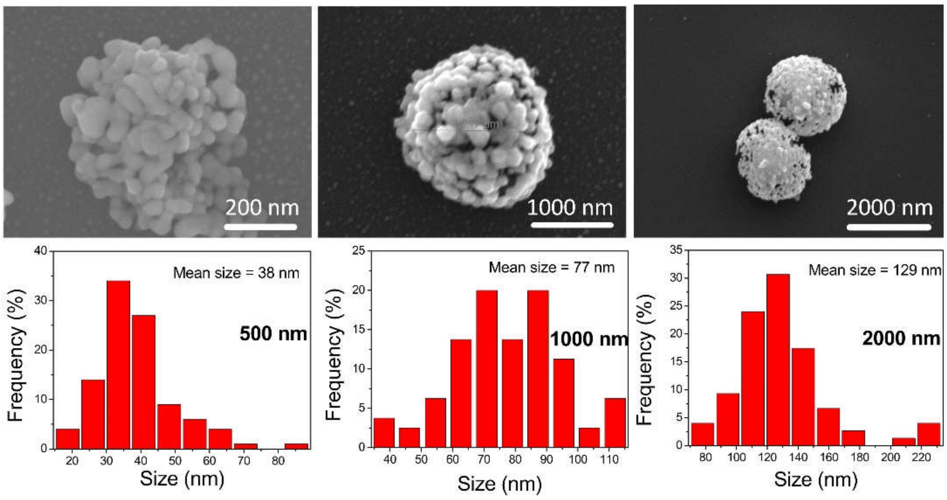

Figure S3. Typical SEM images (upper) and size distributions (lower) of nanoparticles formed on 500 nm, 1000 nm and 2000 nm silver nanoclusters. The mean sizes of nanoparticles on nanoclusters are 38 nm, 77 nm and 129 nm, respectively.

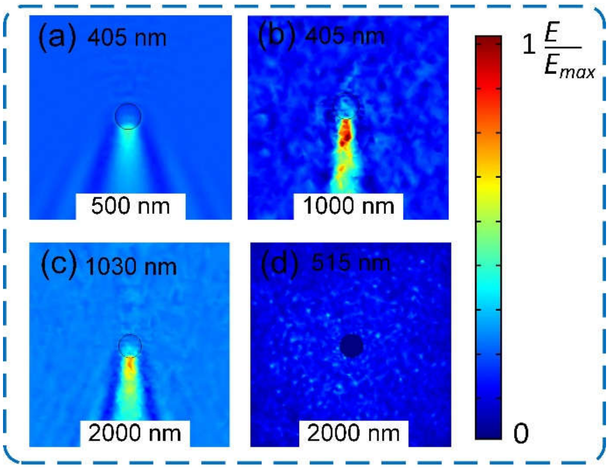

Figure S4. The propagation of laser through the silica microsphere with (**a**) a diameter of 500 nm and incident wavelength of 405 nm; (**b**) a diameter of 1000 nm and incident wavelength of 405 nm; (**c**) a diameter of 2000 nm and incident wavelength of 1030 nm; and (**d**) a diameter of 2000 nm and incident wavelength of 515 nm.

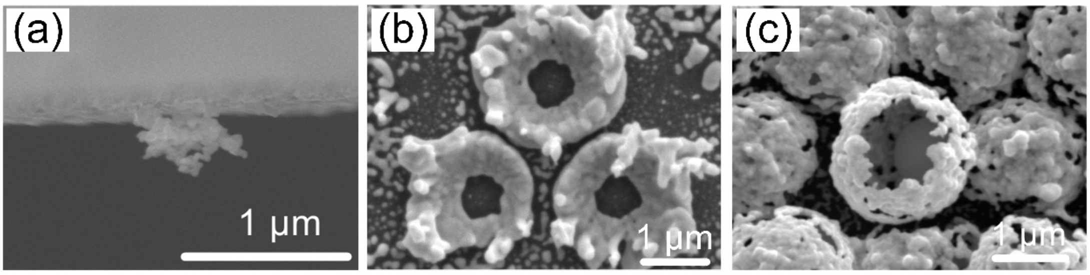

Figure S5. SEM images of (**a**) the nanocluster from cross sectional view; (**b**) bowl and (**c**) hollow structures of the nanoclusters after eliminating silica microspheres using the hydrofluoric acid.

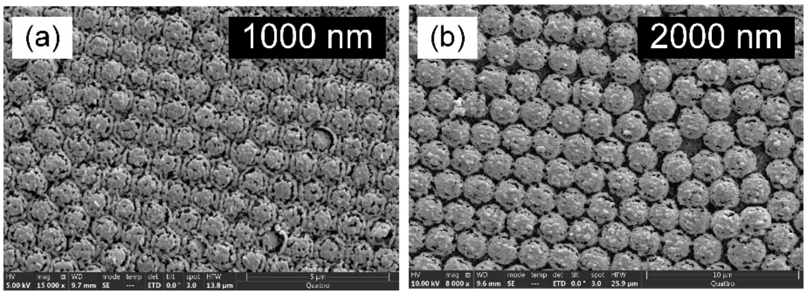

Figure S6. Plasmonic superstructure arrays with periods of (**a**) 1000 nm and (**b**) 2000 nm fabricated using a femtosecond laser with wavelengths at 515 nm and 1030 nm, respectively.

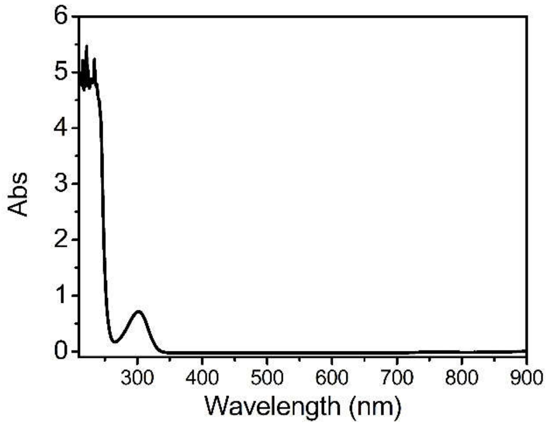

Figure S7. Absorption spectrum of silver precursor solution.

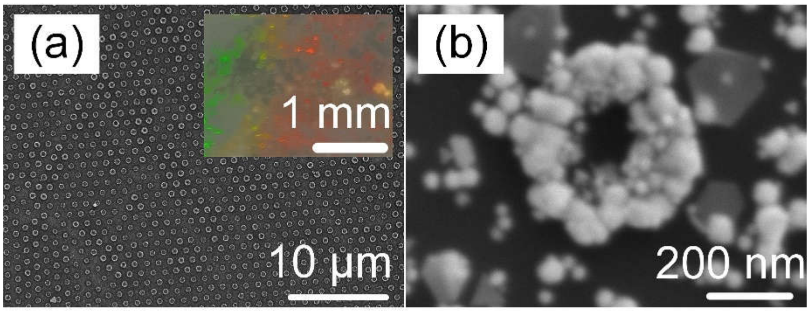

Figure S8. (**a**) Large area image of gold plasmonic superstructure arrays with a period of 1 µm fabricated by laser near-field reduction. (**b**) Single gold nanocluster. The gold precursor was used for irradiation with second harmonic 515 nm femtosecond laser near-field reduction.

The gold precursor consists of 7.5 mg hydrogen tetrachloroaurate tetrachydrate ($HClO_4 \cdot 4H_2O$), 56 mg polyvinylpyrrolidone (PVP, K-90), 2 mg trisodium citrate dehydrate in 1.5 ml H_2O. The second harmonic 515 nm femtosecond laser is generated by a lithium triborate (LBO) crystal. The laser energy density is set at 0.35 nJ/mm^2 and the repetition rate is 1008 kHz.

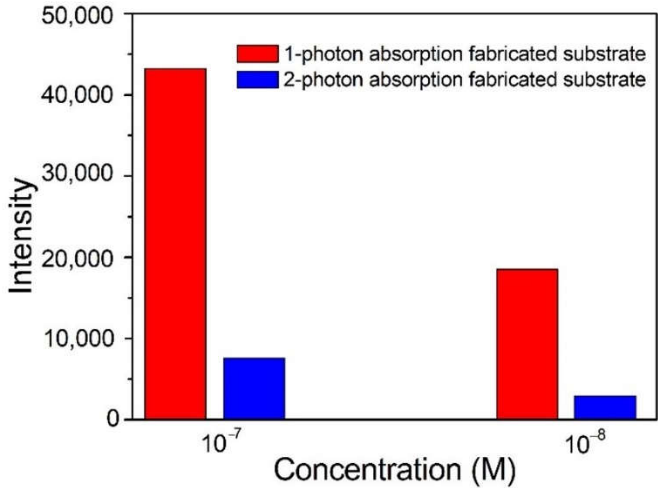

Figure S9. SERS intensities of R6G molecules with two different concentrations at the Raman peak of 610 cm^{-1} using the plasmonic superstructure arrays fabricated by singe- (red) and two-photon (blue) absorption. The Raman excitation wavelength is 633 nm.

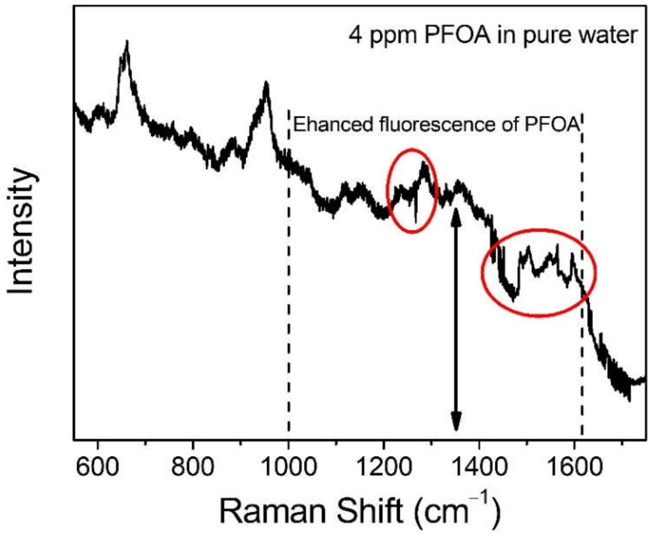

Figure S10. Raman spectrum of 4 ppm PFOA in pure water on superstructure array. The measurement conditions: excitation power and exposure time were set at 2.8 mW and 5 s with 2 accumulations.

To directly measure the Raman peaks of PFOA, the excitation condition should be changed from that for CV + PFOA, specifically, the excitation power increased from 1.4 mW to 2.8 mW and exposure time, from 1s to 5s. However, with increase of both the excitation power and the exposure time, the accumulated fluorescence will be also increased. In fact, the increment of fluorescence is much higher than Raman scattering. The enhanced fluorescence will influence on the accuracy of the Raman results. For example, we directly measured 4 ppm PFOA in pure water using silver superstructure as shown in Figure S10. The enhanced fluorescence of PFOA was figured out at the regions between two dash lines where the heights of Raman peaks are reduced due to the fluorescence, causing the difficulties for the Raman peak identification. Moreover, strong fluorescence will also give rise to the fluctuation of Raman signals, leading to the inaccuracy of the Raman results, which is obvious at the two regions labeled in Raman spectrum with red circles. The sudden drops of Raman intensities can be found in these two regions due to the fluorescence. This fluctuation may erase the Raman peaks of PFOA and provides the incorrect results. Therefore, an indirect way using CV solution mixed with PFOA and detecting CV was adopted to analyze PFOA, instead of direct detection of PFOA.

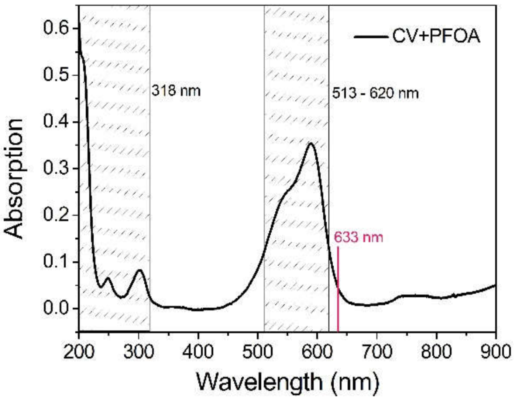

Figure S11. The absorbance of crystal violet (CV) solution mixed with PFOA.

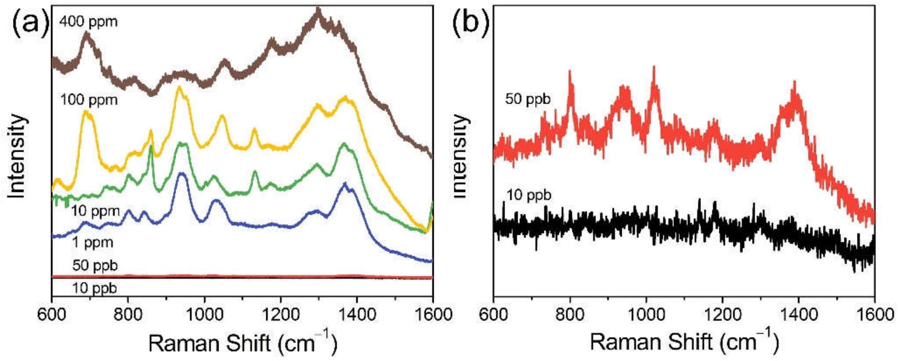

Figure S12. (**a**) SERS spectra of the 10^{-7} M crystal violet mixed with different concentrations of PFOA measured using the plasmonic superstructure arrays with a period of 1000 nm. (**b**) The magnified SERS spectra (**a**) of 10^{-7} M crystal violet mixed with PFOA of 50 ppb (red) and 10 ppb (black). The Raman excitation wavelength is 785 nm with a excitation laser power of 8 mW.

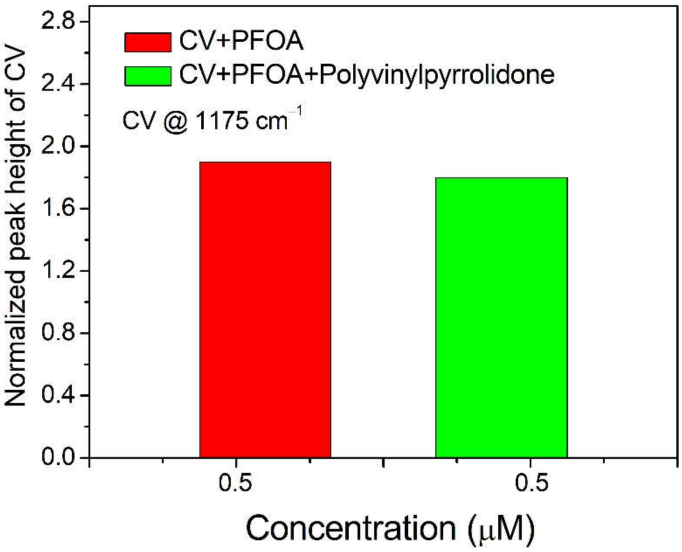

Figure S13. A comparison of Raman intensity at 1175 cm^{-1} for CV + PFOA (5×10^{-7} M) solution (red) and that mixed with polyvinylpyrrolidone (5×10^{-7} M) (green). Concentration of CV was fixed at 10^{-7} M. The peak height was normalized by the peak height of pure CV solution.

Reference

1. Johnson P.B., Christy R.W. Optical constants of the noble metals. *Phys. Rev. B* **1972**, *6*, 4370–4379.
